# Going Bone Deep: Osseous Rosai–Dorfman Disease in an Adult with Recurrent, Culture-Negative Osteomyelitis

**DOI:** 10.1155/2018/6151738

**Published:** 2018-05-17

**Authors:** Dominick S. DeFelice, Megan L. Srinivas, Sara E. Wobker, Jonathan B. Parr

**Affiliations:** ^1^School of Medicine, University of North Carolina at Chapel Hill, Chapel Hill, NC, USA; ^2^Department of Medicine, Division of Infectious Diseases, School of Medicine, University of North Carolina at Chapel Hill, Chapel Hill, NC, USA; ^3^Department of Pathology and Laboratory Medicine, School of Medicine, University of North Carolina at Chapel Hill, Chapel Hill, NC, USA

## Abstract

A patient presented for medical care on three separate occasions over the course of two years with recurrent right knee pain attributed to chronic osteomyelitis. Careful assessment revealed that his symptoms were caused by osseous Rosai–Dorfman disease. This case presents an alternative diagnostic possibility for culture-negative chronic osteomyelitis.

## 1. Introduction

Rosai–Dorfman disease (RDD), also known as sinus histiocytosis with massive lymphadenopathy, is a rare, nonmalignant histiocytic proliferative disease first described in 1969[1]. While it typically presents within the first several decades of life with enlarged cervical lymph nodes and fever, its primary osseous form can be indolent and difficult to distinguish from osteomyelitis [2]. The etiology of RDD is unknown, and diagnosis requires careful pathological evaluation of the involved tissue. Regardless of the site involved, most cases of RDD spontaneously regress months to years following diagnosis [3, 4].

## 2. Case Report

The patient was a 46-year-old male with a history of hypertension and obesity who presented for scheduled excision and curettage of a portion of the right proximal tibia for presumed chronic osteomyelitis. He initially presented to care 2.5 years ago after developing acute, localized pain, warmth, and swelling of the right knee after moving furniture. He received a corticosteroid knee injection for a presumed structural process and achieved symptomatic relief. Synovial fluid analysis was not performed.

One year later, his symptoms returned, and he was diagnosed with osteomyelitis of the right proximal tibia based on biopsy and imaging. Plain radiographs of the leg showed a lytic bone lesion, and bone biopsy histology demonstrated necrosis with acute inflammation. He was afebrile and had an elevated C-reactive protein (CRP) of 13.9 mg/L (normal range: <10 mg/L) and erythrocyte sedimentation rate (ESR) of 30 mm/h (normal range: 0–22 mm/h), but normal white blood cell count (WBC) of 7.2 × 10^9^/L and otherwise unremarkable complete blood count and basic metabolic panel. He underwent right proximal tibia excision and curettage with vancomycin and tobramycin antibiotic bead grafting. Intraoperative findings included bony tissue that was grossly abnormal and malodorous. Intraoperative bone cultures were negative. He completed an 8-week course of empirical vancomycin, ciprofloxacin, and metronidazole for culture-negative osteomyelitis, with normalization of his CRP and ESR at the end of therapy.

**Figure 1:**
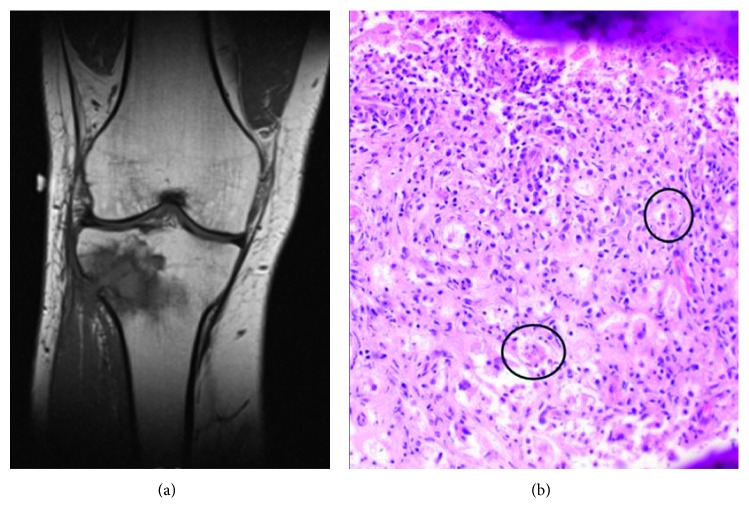
(a) Coronal MRI demonstrating a T1-hypointense lesion in the right proximal tibia with cortical destruction; (b) hematoxylin and eosin- (H&E-) stained section of the patient's tibia lesion demonstrating numerous eosinophilic histiocytes and emperipolesis (black circles).

His symptoms initially resolved after surgery. An X-ray obtained one month later demonstrated minimally increased sclerosis of the proximal tibia, which could be consistent with postsurgical changes, without evidence of progression of the lesion. Four months later, however, his right knee pain, swelling, and impaired ambulation returned. He denied trauma, unusual exposures, and IV drug use. He underwent magnetic resonance imaging (MRI) which demonstrated progression of osteomyelitis with abscess formation, cortical breakthrough, and involvement of the surrounding soft tissue (Figure 1(a)). His CRP and ESR were elevated at 24.6 mg/L and 31 mm/h, respectively, but his WBC of 4.0 × 10^9^/L remained normal. Due to concerns about recurrent, progressive osteomyelitis, he underwent a second excision and curettage of the right proximal tibia. Operative findings were again notable for grossly abnormal, friable material at the involved site. Empirical intravenous vancomycin and piperacillin-tazobactam were initiated postoperatively, but intraoperative bone cultures and blood cultures were negative. As a result, the patient faced the possibility of another extended course of empirical antibiotics followed by an above-the-knee amputation if his symptoms recurred.

Careful review of his bone biopsy by pathology yielded a different diagnosis. Findings again included mixed inflammatory cells, but now also involved abundant foamy histiocytes and eosinophilic histiocytes with emperipolesis, a phenomenon observed when other inflammatory cells (e.g., neutrophils, plasma cells, and lymphocytes) pass through the cytoplasm of a histiocyte (Figure 1(b)). The bone stained negative for fungi using Grocott's methenamine silver (GMS) and acid-fast bacilli (AFB). The cells of interest were stained for CD68, S100, and CD1a. CD68 and S100 were positive in the histiocytes, with negative CD1a, supporting a diagnosis of RDD.

His antibiotics were discontinued upon diagnosis, and he was discharged home. A body positron emission tomography-CT performed shortly after surgery to evaluate for other potential areas of disease was negative, and his knee pain and swelling improved initially after surgery. However, eight months later, he presented to hematology and oncology clinic with a fourth recurrence of symptoms, for which localized radiation therapy was recommended.

## 3. Discussion

This report describes the clinical course of a patient who presented on three separate occasions with localized right knee inflammatory symptoms that were initially presumed to be due to infection but eventually proven to be osseous Rosai–Dorfman disease. During the workup for the second and third instances of his localized right knee inflammatory symptoms, he had findings suggestive of osteomyelitis: elevated inflammatory markers, a lytic bone lesion, and inflamed, necrotic bone material on pathology.

In hindsight, two aspects of his initial clinical course had actually pointed away from osteomyelitis. First, repeated blood and bone cultures, including aerobic and anaerobic bacterial, fungal, and AFB cultures, failed to reveal an organism. Second, he had no risk factors for osteomyelitis other than corticosteroid knee injection, which was performed after the initial development of symptoms. Additionally, he lacked typical risk factors such as diabetes, immunodeficiency, trauma, indwelling hardware, or peripheral vascular disease. Thus, we began to entertain the possibility of a noninfectious etiology.

RDD typically presents in pediatric patients with enlarged lymph nodes, fever, elevated WBC, and elevated ESR. While the classic presentation involves profound cervical lymphadenopathy, approximately 40% of cases involve extranodal sites [5]. The etiology of RDD is unknown, but both infectious and immunologic causes have been proposed. RDD most commonly affects lymph nodes, but secondary extranodal involvement of skin, the upper respiratory tract, and bone occurs frequently. However, primary osseous involvement is rare, occurring in less than 10% of cases [2, 6]. In a recent case series of osseous RDD, the cranium (31%), facial bones (22%), and tibia (18%) were most commonly affected, followed by the spine/sacrum, femur, and pelvis. Additionally, primary osseous disease often occurred in adulthood (mean age 31 years with a standard deviation of 20 years) and typically involved only one or two bones [2]. We diagnosed our patient with relapsing, primary osseous RDD given the lack of lymphadenopathy or other extranodal findings.

In addition to RDD, the differential diagnosis for recurrent, sterile osteomyelitis should include other uncommon causes such as Langerhans' cell histiocytosis (LCH) and related histiocytoses [7]. LCH represents a collection of clinically variable disorders that can cause lytic bone lesions and overlying soft tissue pain in children and adults. However, LCH is a disorder of neoplastic histiocytic proliferation that can be distinguished from RDD based on its more severe course and the presence of Langerhans' cells on pathology, described as CD1a-positive cells that exhibit “racquet-shaped” granules on electron microscopy [8].

Our patient underwent repeated imaging, debridement, and treatment with empirical antibiotics before receiving a definitive diagnosis. Clinicians often make the diagnosis of osteomyelitis without bone biopsy, the diagnostic gold standard. When the clinical history suggests osteomyelitis, other findings can often be used to support diagnostic decisions, including the ability to visualize or probe to bone, an elevated ESR and/or CRP, cortical destruction on X-ray, or bone marrow changes on MRI. However, attention to the possibility of noninfectious etiologies is important, especially in cases where the patient has no risk factors, bone pathology and culture fail to reveal organisms, and the patient does not adequately respond to therapy. Common noninfectious causes include malignancy (e.g., lymphoma, multiple myeloma, metastatic disease, or primary bone cancers) and hyperparathyroidism, but atypical causes such as RDD and LCH should also be considered.

RDD is associated with a benign outcome in most cases but can occasionally lead to a relapsing and remitting course and, rarely, death [5]. With treatment, total or partial remission of osseous RDD is seen in nearly 80% of cases [2]. Two effective treatment approaches have been reported: (1) surgical debulking or radiotherapy to alleviate compression of vital organs and (2) steroids when fever without infection and/or large lymph nodes are present [3]. Treatment of cases with acyclovir, interferon, anthracyclines, *Vinca* alkaloids, or other alkylating agents has been reported, but evidence supporting their use is limited. However, a recent report of RDD with an activating mutation of KRAS, a human proto-oncogene, that responded to treatment with cobimetinib suggests there may be a role for targeted therapy using MAPK kinase (MEK) inhibitors [9].

Our patient underwent two debridements, which served as “debulking” procedures, with resulting symptomatic improvement. While it is possible that his disease might have resolved without debulking, his clinical course alongside other case reports suggests that surgical intervention has a role in RDD management [4, 10]. His subsequent recurrence, however, highlights the relapsing nature of some cases of RDD despite intervention. The prognosis of his individual case is unknown, given the paucity of published reports of RDD.
